# Persistence of *Candida albicans* in the Oral Mucosa Induces a Curbed Inflammatory Host Response That Is Independent of Immunosuppression

**DOI:** 10.3389/fimmu.2019.00330

**Published:** 2019-02-27

**Authors:** Florian R. Kirchner, Katharina Littringer, Simon Altmeier, Van Du T. Tran, Franziska Schönherr, Christina Lemberg, Marco Pagni, Dominique Sanglard, Nicole Joller, Salomé LeibundGut-Landmann

**Affiliations:** ^1^Section of Immunology, Vetsuisse Faculty, University of Zurich, Zurich, Switzerland; ^2^Institute of Experimental Immunology, University of Zurich, Zurich, Switzerland; ^3^Vital-IT Group, Swiss Institute of Bioinformatics, Lausanne, Switzerland; ^4^Institute of Microbiology, University of Lausanne and University Hospital Center, Lausanne, Switzerland

**Keywords:** *Candida albicans*, oropharyngeal candidiasis, immune regulation, persistence, IL-17, IL-10, regulatory T cells

## Abstract

Controlled immune activation in response to commensal microbes is critical for the maintenance of stable colonization and prevention of microbial overgrowth on epithelial surfaces. Our understanding of the host mechanisms that regulate bacterial commensalism has increased substantially, however, much less data exist regarding host responses to members of the fungal microbiota on colonized surfaces. Using a murine model of oropharyngeal candidiasis, we have recently shown that differences in immune activation in response to diverse natural isolates of *Candida albicans* are associated with different outcomes of the host-fungal interaction. Here we applied a genome-wide transcriptomic approach to show that rapid induction of a strong inflammatory response characterized by neutrophil-associated genes upon *C. albicans* colonization inversely correlated with the ability of the fungus to persist in the oral mucosa. Surprisingly, persistent fungal isolates showed no signs of a compensatory regulatory immune response. By combining RNA-seq data, genetic mouse models, and co-infection experiments, we show that attenuation of the inflammatory response at the onset of infection with a persistent isolate is not a consequence of enhanced immunosuppression. Importantly, depletion of regulatory T cells or deletion of the immunoregulatory cytokine IL-10 did not alter host-protective type 17 immunity nor did it impair fungal survival in the oral mucosa, indicating that persistence of *C. albicans* in the oral mucosa is not a consequence of suppressed antifungal immunity.

## Introduction

Opportunistic infections with fungi are an increasing cause of morbidity and mortality worldwide. *C. albicans* is one of the most important disease-causing fungi in humans. It is found as a commensal in the human gastrointestinal and genital tracts with a large proportion of healthy individuals being carriers, but it may become pathogenic under certain conditions. Disease symptoms range from mild to severe superficial infections of the oral and vaginal mucosae, the skin and the nails, affecting millions of people worldwide (1–5). More rarely, *C. albicans* causes systemic diseases associated with high mortality rates (6). The development of *C. albicans* mucosal infections is mainly attributed either to defects in host cellular immunity, including those resulting from primary or secondary immunodeficiency, or to changes in the normal microbiota that may be caused by antibiotic treatment (3, 5). Defects in epithelial barrier integrity are also associated with infections, highlighting the importance of intact epithelial function for preventing fungal entry into the tissue (7). In addition to host factors, *C. albicans* infections may be favored by increased expression of virulence attributes of the fungus. Genetic variations within the species of *C. albicans* that result in phenotypic diversity within the fungus have been found to modulate its pathogenicity at epithelial surfaces and systemically (8–12). The decision between *C. albicans* commensalism and disease is thus the result of a fine balance between fungal virulence and host defense mechanisms.

The experimental model of oropharyngeal candidiasis (OPC) in mice has been widely used to study the interaction of *C. albicans* with the host at mucosal surfaces *in vivo* (13). It allowed unraveling antifungal immune mechanisms against *C. albicans*, such as the interleukin 17 (IL-17)-pathway (14), and to explore the pathogenicity of *C. albicans* mutants at the mucosal surface (15, 16).

Using the common laboratory strain SC5314 for infection in this model only partially reflects the situation of OPC in humans (where *C. albicans* is a commensal). In wild type mice, this highly virulent *C. albicans* isolate is rapidly cleared from the oral mucosa (17). However, we have recently shown that depending on the fungal isolate, the murine oral mucosa can be colonized with *C. albicans* for a prolonged period of time without immunosuppression of the host (12), thus mimicking the situation in humans.

Our initial investigations of the differences between acute and persistent OPC in mice have revealed major differences in the host response that was induced by different isolates of *C. albicans* (12). Most strikingly, the differential degree of inflammation that was triggered by virulent strains (such as strain SC5314) compared to persistent strains (such as strain 101) correlated with the differential outcome of infection (12). However, the basis of the differential host response triggered by different strains remains unclear.

Here, we applied an unbiased genome-wide approach to obtain a comprehensive view on the host response to two functionally divergent fungal strains and to assess differences in the host response that might modulate the balance between fungal persistence and rapid clearance. Moreover, we addressed whether persistent *C. albicans* had a propensity to trigger an immunosuppressive response in the host that might be responsible for the curbed immune activation and in turn allow prolonged colonization in the host.

## Materials and Methods

### Mice

*Foxp3*-GFP.KI x *Il10*-Thy1.1 reporter mice (18, 19), DEREG mice (20) and *Il10*^−/−^ mice (21) were obtained from Vijay Kuchroo (Brigham and Women's Hospital, Harvard Medical School, Boston, MA, USA), Manfred Kopf (ETH Zurich, Switzerland), and the Swiss Immunological Mouse Repository, respectively and bred at the Laboratory Animal Service Center (University of Zürich, Switzerland). Wildtype (WT) C57BL/6J mice were purchased from Janvier Elevage. All mice were kept in specific pathogen-free conditions and used in sex- and age-matched groups at 6–15 weeks of age. DEREG mice were treated with 1 μg diphtheria toxin i.p. on day 11 and 13 post-infection. Where indicated, 0.125 mg anti-CD25 antibody (clone PC-61.5.3, BioXCell) or the corresponding isotype control were administered i.p. per mouse 7 days prior to infection.

### Fungal Strains and OPC Infection Model

*C. albicans* strains SC5314 (22) and 101 (12) were grown in YPD medium at 30°C and 180 rpm for 15–18 h. Mice were infected sublingually with 2.5 × 10^6^ *C. albicans* yeast cells as described (23), without immunosuppression. In co-infection experiments, mice were infected with 1.25 × 10^6^ yeast cells of each strain, i.e., with a total of 2.5 × 10^6^ yeast cells. For determination of the fungal burden, the tongue of euthanized animals was removed, homogenized in sterile 0.05% NP40 in H_2_O for 3 min at 25 Hz using a Tissue Lyzer (Qiagen) and serial dilutions were plated on YPD agar containing 100 μg/ml Ampicillin. Infection of mice with strain 101 resulted in detectable fungal loads in the tongue and in the feces for >60 days, in some mice >1 year.

### Preparation of Tongue Epithelial Sheets and RNA Isolation

The tongue was cut in half to obtain the dorsal part, which was then freed from muscle tissue with a scalpel and floated on PBS containing 2.86 mg/ml dispase II (Roche) for 60 min with the epithelial side facing upwards to separate the epithelial tissue from the lamina propria. Epithelial sheets were incubated in RNAlater (Sigma-Aldrich) for 1 min immediately after isolation and then grinded in liquid N_2_. Three epithelial sheets were pooled for the generation of each RNA-seq replicate and two replicates were generated.

RNA isolation from epithelial sheets was done by combining two phase separations and DirectZol™ RNA MiniPrep kit (Zymo Research) as described (24). DNase treatment was performed off-column using the DNA-free™ Kit (Life Technologies). RNA integrity was determined using a 2100 Bioanalyzer system (Agilent Technologies) according to the manufacturer's instructions. Samples were only included in the study if the RNA integrity value (RIN) was above 7.5 and no obvious degradation was observed.

### Preparation of cDNA Libraries and Sequencing

cDNA libraries were generated using the SureSelect multiplexed sequencing kit with strand-specific RNA library preparation for Illumina (Agilent Technologies) according to the manufacturer's instructions. In brief, mRNA was purified using poly(A) beads, fragmented and double-stranded cDNA with ligated adapters was generated. The library was amplified using primers that match the adapters. During this step, RNA-seq indexes were inserted into the libraries for multiplexing. The collected double-strand cDNA was then amplified and indexed in a separate PCR. RNA quality, fragment size, and cDNA concentration were determined using a fragment analyzer automated CE system (Advanced Analytical) and a Qubit fluorometer (Invitrogen).

cDNA libraries were subjected to cluster generation using the Illumina TruSeq PE cluster kit v3 reagents and sequenced on the Illumina HiSeq 2500 system with TruSeq SBS kit v3 reagents at the Lausanne Genomic Technologies Facility (LGTF).

### RNA-Seq Data Analysis

RNA-seq purity-filtered reads were adapter- and quality-trimmed with *cutadapt* (v1.2.1) (25) and filtered for low complexity with *seq_crumbs* (v0.1.8) (https://bioinf.comav.upv.es/seq_crumbs). After alignment against the mouse genome GRCm38.p4 using STAR (v2.4.2a), *htseq-count* (v0.5.4p3) (26) was used to summarize the number of read counts per gene locus. Genes with counts fewer than one per million in all samples were removed from the statistical analysis, yielding 13,855 remaining genes. Data normalization and differential expression analysis were performed in R (v3.2.2) as follows. Normalization of read count data was performed with the *edgeR* package using the TMM (trimmed mean of M-values) method (27). Normalized read count data was transformed to log2-counts with the *voom* transformation (28). A linear model was applied to the transformed data using the *limma* package (29) on all conditions, all in duplicates. The contrasts representing the difference between the infected and naïve conditions were studied, including conditions at 9 h, 1 day, 3 days, 7 days post-infection with strains SC5314 and 101, respectively. *P*-values produced from the differential analysis were adjusted using Benjamini & Hochberg correction (30). Genes were considered to be differentially regulated if their expression was altered by a factor of at least 2-fold with adjusted *p*-values < 0.05 (false discovery rate, FDR). Gene Ontology (GO) enrichment analysis was done with the *topGO* package (version 2.32.0) using the *fisher* statistic and *weight01* algorithm. This analysis was restricted to all GO terms that are offspring of GO:0002376 (immune system processes) and to genes that are differentially regulated in at least one contrast. An additional enrichment analysis was performed with the *MetaCore*^*TM*^ software (Thomson Reuters, https://clarivate.com/products/metacore) on all GO terms belonging to biological process. Heat maps and hierarchical clustering were generated with the *Morpheus* software (Broad Institute, https://software.broadinstitute.org/morpheus) using the distance of one minus Pearson correlation and the average linkage mode. The data analyzed here are accessible under the NCBI BioProject accession number PRJNA491801.

### RNA Isolation From Total Tongue and Quantitative RT-PCR

Isolation of total RNA from bulk tongues was carried out according to standard protocols using TRI Reagent® (Sigma Aldrich). cDNA was generated by RevertAid reverse transcriptase (Thermo Fisher). Quantitative PCR was performed using SYBR Green (Roche) and a QuantStudio 7 Flex (Life Technologies) instrument. The primers used for qPCR are listed in Table S1. All qRT-PCR assays were performed in duplicate and the relative expression (rel. expr.) of each gene was determined after normalization to β-actin transcript levels.

### Preparation of Lymph Node Cells for T Cell and Treg Analysis by Flow Cytometry

Cervical lymph nodes were removed and single cell suspensions were prepared by digestion with DNase I (2.4 mg/ml, Roche) and Collagenase I (2.4 mg/ml, Invitrogen) in PBS for 15 min at 37°C. For inducing cytokine production by primed T cells, 10^6^ cervical lymph node cells were re-stimulated for 6 h with 1 × 10^5^ MutuDC1940 cells (31) pulsed with 2.5 × 10^5^/ml heat-killed *C. albicans* or left unpulsed. Brefeldin A (10 μg/ml, AppliChem) was added for the last 5 h to inhibit the secretory pathway. Intracellular IL-17 and IFN-γ production was then analyzed by flow cytometry.

### Preparation of Tongue Cells for Flow Cytometry

For quantification of neutrophils, tongues were removed and digested with DNase I (2.4 mg/ml, Roche), and Collagenase IV (4.8 mg/ml) in PBS for 45 min at 37°C. For analysis of Tregs, mice were anesthetized with a sublethal dose of Ketamin (100 mg/kg), Xylazin (20 mg/kg), and Acepromazin (2.9 mg/kg) and perfused by injection of PBS into the right heart ventricle prior to removing the tongue. Tongues were cut in half and the underlying muscle tissue was carefully removed using a scalpel. The remaining tongue tissue was cut into small pieces and digested with DNase I (2.4 mg/ml, Roche), Collagenase IV (2.4 mg/ml) and in some case Trypsin (1 mg/ml) in PBS for 45 min at 37°C. Single cell suspensions were obtained by passing the digested tissue through a 70 μm strainer using icecold PBS supplemented with 1% FCS and 2 mM EDTA and then stained for flow cytometry.

### Flow Cytometry

Single cell suspensions of tongue and lymph nodes were stained in PBS supplemented with 1% FSC, 5 mM EDTA, and 0.02% NaN_3_. LIVE/DEAD Near IR stain (Life Technologies) was used for exclusion of dead cells. The antibodies for surface and intracellular cytokine staining are listed in Table S2. For intracellular cytokine staining, cells were fixed and permeabilized using BD Cytofix/Cytoperm reagent (BD Biosciences) and subsequently incubated in Perm/Wash buffer (BD Biosciences). All extracellular and intracellular staining steps were carried out on ice.

For intranuclear Foxp3 staining, cells were fixed/permeabilized for 40 min at RT using the Foxp3 Staining Buffer Set (eBioscience) and subsequently stained for 40 min at RT in BD Perm/Wash buffer (BD Biosciences), Cells were acquired on a FACS Gallios (Beckman Coulter), a SP6800 Spectral Analyzer (Sony) or a BD LSRFortessa (BD Biosciences). The data were analyzed with FlowJo software (FlowJo LLC). The gating of the flow cytometric data was performed according to the guidelines for the use of flow cytometry and cell sorting in immunological studies (32), including pre-gating on viable and single cells for analysis. Absolute cell numbers of lymphoid cell populations were calculated based on a defined number of counting beads (BD Biosciences, Calibrite Beads), which were added to the samples before flow cytometric acquisition.

### Histology

For histology, tissue was fixed in 4% PBS-buffered paraformaldehyde overnight and embedded in paraffin. Sagittal sections (9 μm) were stained with Periodic-acidic Schiff (PAS) reagent and counterstained with Haematoxilin and mounted with Pertex (Biosystem, Switzerland) according to standard protocols. Images were acquired with a digital slide scanner (NanoZoomer 2.0-HT, Hamamatsu) and analyzed with NDP.view2.

## Results

### The Host Response to *C. albicans* Strain 101 Is Delayed Compared to Strain SC5314

To obtain genome-wide information about the host response to *C. albicans* in the oral mucosa, we performed a transcriptomic analysis. We used the two functionally distinct *C. albicans* strains SC5314 and 101 to assess strain-specific differences between acute and persistent infection in mice at 9 h, 1 day, 3 days and 7 days post-infection. To enrich for host tissue in direct contact with the fungus, we isolated epithelial sheets from the tongue by dispase II-mediated digestion of the basal membrane. Epithelial sheets were strongly enriched for epithelial cells compared to the proportion found in the bulk tongue, but they also contained tissue-resident and infiltrated immune cells as evidenced by histology and flow cytometry (Figure S1).

**Figure 1:**
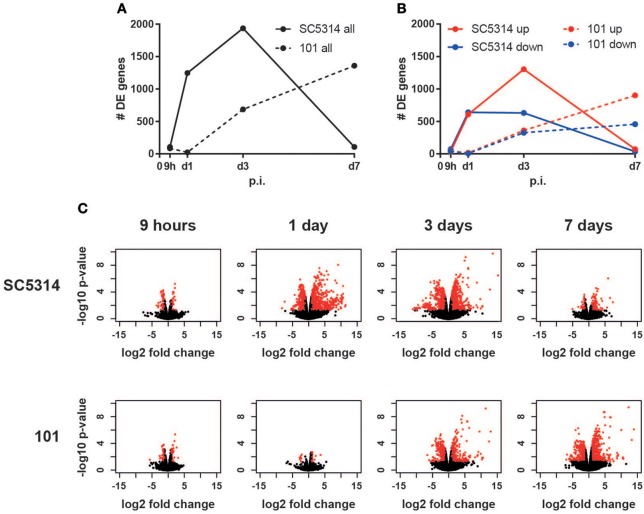
The host response to *C. albicans* strain 101 is delayed compared to strain SC5314. Epithelial sheet from *C. albicans* strain SC5314- and strain 101-infected WT mice were subjected to RNA-seq analysis. **(A)** Graph showing the total number of differentially expressed (DE) genes at the indicated time points compared to naïve controls. **(B)** Separate display of the up- and down-regulated genes at the indicated time points. **(C)** Volcano plots displaying the fold changes and the FDR of all genes detected in each condition separately. Genes with FDR < 0.05 and fold change < −2 or > 2 are marked in red.

Epithelial sheets were subjected to RNA isolation, cDNA library generation and sequencing. Analysis of the number of differentially expressed genes over time in comparison to uninfected conditions revealed a rapid onset of the transcriptional response to the virulent strain SC5314 between 9 and 24 h post-infection, whereas a slower kinetic was observed after infection with the persistent strain 101, which started rising after day 1 post-infection and increased continuously until day 7 post-infection (Figures 1A–C). Interestingly, in case of strain SC5314, the number of differentially expressed genes increased until day 3 post-infection (Figures 1A–C), even though the fungal burden is known to decline rapidly at this time point for this specific isolate (33). The genes differentially expressed on day 3 post-infection largely differed from those regulated earlier (Figure S2). Finally, by day 7, only very few genes were changed in comparison to uninfected controls (Figures 1A–C, Figure S2) indicating that homeostasis was rapidly restored after clearance of strain SC5314.

### Quantitative and Qualitative Differences in the Host Response to Virulent and Persistent OPC

Given the delayed response observed to the persistent strain 101 compared to the virulent strain SC5314, we were interested whether the two responses were qualitatively comparable, regardless of kinetics of the observed changes. While a large number of genes were selectively regulated by SC5314, there was also a considerable overlap between the two responses, whereby most co-regulated genes were delayed by one to two time points in case of strain 101 (Figure S2). Likewise, at the level of the regulated biological processes, as defined at the level of gene ontology, there was a good concordance of the response to the two fungal strains, again with slower kinetics and lower FDR in case of strain 101 (Figure S3). The processes that were most strongly induced early after infection comprised those linked to antimicrobial response and immunity, while on day 3 post-infection with strain SC5314, metabolic processes dominated. The processes that were most strongly repressed by both strains comprised those linked to development and negative regulation of signaling (Figure S3).

**Figure 2:**
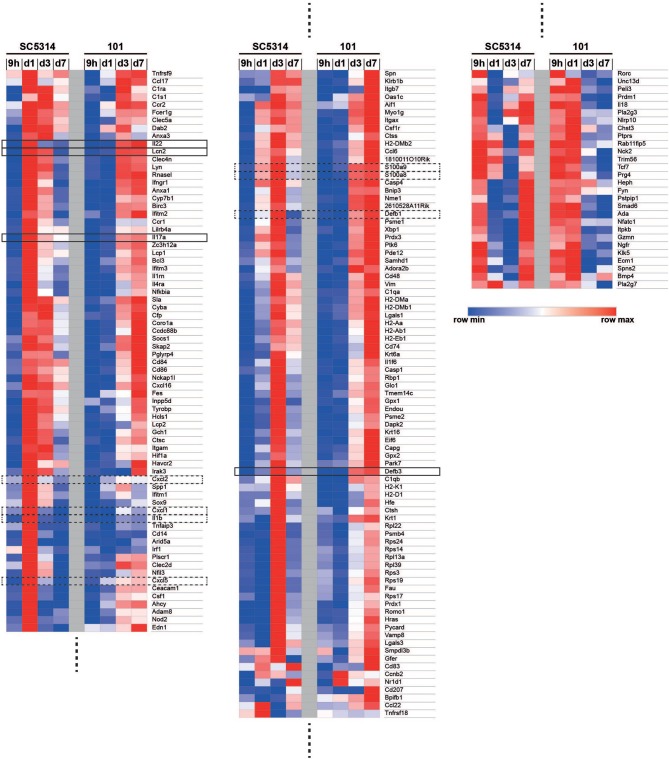
Co-regulated and differentially regulated genes in the mucosal response to strains 101 and SC5314. The GO term enrichment analysis on genes that are differentially regulated in at least one contrast for the 62 GO terms that are offspring of GO: 0002376 (immune system processes) with *p* < 0.05 yielded 182 genes. Genes were ordered by hierarchical clustering of their log2 fold changes, which are displayed in a heat map with a row-scaling color scheme.

Among the immune system processes that were most significantly regulated by strain SC5314, some were found to have a comparably high FDR in the response to strain 101, including GO terms linked to chemotaxis and cell migration. This strain-specific difference was also reflected at the level of the genes that were annotated to these processes: genes encoding for the neutrophil chemokines CXCL1, CXCL2, and CXCL5 as well as IL-1β were strongly induced in response to SC5314 on day 1 post-infection, but very poorly in response to 101 at any time point analyzed (Figure 2). Even after 60 days of infection, when mice were still highly colonized with strain 101, expression of these factors remained as low as in uninfected controls (Figures S4A–B). This was consistent with the curbed recruitment of neutrophils to the tongue infected with strain 101 in comparison to strain SC5314 (12). In some mice, strain 101 persisted for over 1 year without causing inflammation or triggering neutrophil recruitment (Figures S4C–E).

Among the genes annotated to immune system processes that were significantly regulated (FDR < 0.05), we observed many that were regulated to a comparable degree by both fungal strains, including genes associated with the IL-17 pathway (Figure 2). Conserved induction of the IL-17 pathway by both fungal strains was also confirmed at the level of IL-17 target genes, including those coding for S100a8, S100a9, Lipocalin 2, β-defensin-3, and β-defensin-1. They reached highest expression levels on day 1–3 post-infection in case of strain SC5314, and on day 3–7 in case of strain 101, respectively (Figure 2). *Defb3* and *Defb1* genes were an exception to this pattern as their expression did not rise before day 3 in either condition (Figure 2). Overall, this confirmed what we previously observed in response to these two fungal strains (Figure S4F) (12).

### *C. albicans* Strain 101 Does Not Actively Suppress the Host Response at the Onset of Infection

**Figure 3:**
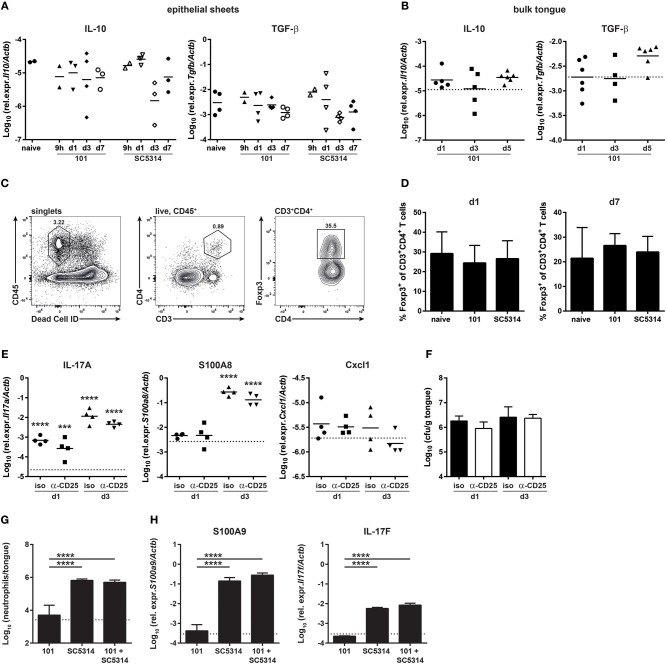
The persistent strain 101 does not suppress the antifungal host response. **(A,B)** Relative expression of *Il10* (left) and *Tgfb1* transcripts (right) in epithelial sheets **(A)** or in bulk tongue tissue **(B)** of mice that were infected with strain 101 or SC5314 for the indicated period of time. Each symbol represents a pool of epithelial sheets from three animals each **(A)** or a single mouse **(B)**. The geomean of each group is indicated. Data are pooled from two independent experiments each. **(C,D)** % Foxp3^+^ Tregs within the viable CD45^+^CD3^+^CD4^+^ population in the tongue of mice that were infected with strain 101 or SC5314 for the indicated period of time. Representative FACS plots and the gating strategy are shown in C, summary plots showing the mean + SD of data pooled from two independent experiments with 3–4 animals per group are shown in D. **(E,F)** Mice were treated with anti-CD25 or isotype control antibody prior to infection with strain 101. Relative expression of *Il17a* (left), *S100a8* (middle), and *Cxcl1* (right) **(E)** and tongue fungal loads **(F)** in the bulk tongue tissue at the indicated time point after infection are shown. In **(E)**, each symbol represents a single animal, The geomean of each group is indicated. The dotted line represents transcript levels in naïve animals (mean of 8 animals). In **(F)**, each bar is the geomean + SD of 4 animals per group. **(G,H)** WT mice were infected sublingually with a 1:1 mixture of strain 101 and strain SC5314 or with each strain alone. Tongues were harvested on day 1 post-infection and analyzed for the infiltration of Ly6G^+^Ly6C^lo^CD11b^+^ neutrophils by flow cytometry **(G)** and for the expression of *S100a9* and *Il17f* **(H)** transcript by RT-qPCR. Data are the mean + SD of 3–4 mice per group. Graphs display data representative of one out of two independent experiments. The dotted line represents the detection limit. Statistics were calculated using one-way ANOVA. In **(E)**, statistics compare infected to naïve groups. ***p < 0.001, ****p < 0.0001.

In search of an explanation for the delayed and more limited host response to strain 101 in comparison to strain SC5314, we examined whether expression of immune regulatory and immunosuppressive genes that might curb the inflammatory response predominated during infection with strain 101. Surprisingly, such genes were not found to be significantly induced in the RNA-seq data set by any of the two strains. Moreover, in a GO process enrichment analysis, processes associated with immune regulation such as “negative regulation of immune response” or “negative regulation of immune system processes” were not altered significantly. This was also consistent with the absence of an increase in IL-10 or TGF-β transcript levels by RT-qPCR in epithelial sheets (Figure 3A). This analysis may not have captured the full spectrum of the antifungal response, as the epithelial sheets used for RNA isolation and sequencing/RT-qPCR do not represent the full complexity of the mucosal immune system and some cells may have been lost during preparation of the epithelial sheets (and others might act at a distance). We therefore quantified IL-10 and TGF-β expression levels in the bulk tongue of 101-infected compared to naïve mice (Figure 3B).

We further looked at regulatory T cells (Tregs) and whether they contribute to suppression of the early host response to strain 101. Immune suppression might be undetectable at the RNA level as transcriptional changes may occur in rare cell populations that are lost in resolution in the whole tissue. Tregs are more abundant in the oral mucosa compared to other organs (34). Detection of lymphocytes in the tongue is challenging due to the small number of cells and the high degree of autofluorescence of this tissue (37, 38). Using a refined protocol of tissue preparation and flow cytometry we were able to detect a small number of Foxp3^+^ T cells in the tongue which were present at comparable frequencies in naïve and infected animals (Figures 3C–D). Moreover, depletion of Tregs prior to infection (Figure S5) did not alter the kinetics of the epithelial response to strain 101 or allow expression of inflammatory genes such as those regulating the neutrophil response (Figure 3E), nor did it affect the fungal load (Figure 3F). This indicates that Tregs are not responsible for the limited and delayed host response at the onset of persistent OPC.

Finally, we tested the capacity of strain 101 to suppress the host response in the oral mucosa with an unbiased approach using a co-infection experiment for which we infected mice with a 1:1 mix of strain 101 and strain SC5314. We questioned whether the presence of strain 101 would lead to a reduced overall host response in the tongue due to a dominant regulatory response. This was however not the case: the induction of a rapid inflammatory response was as efficient after co-infection as after infection with strain SC5314 alone. The recruitment of neutrophils to the site of infection as well as the induction of *Il17f* and *S100a9*, two of the most strongly induced genes by SC5314 on day 1 post-infection, was comparable in both conditions (Figures 3G–H). These data show that strain 101 is not actively repressing the antifungal host-response and that fungal persistence is not a consequence of suppressed immune activation.

### The Treg Compartment Is Normal During Persistent *C. albicans* Infection

**Figure 4:**
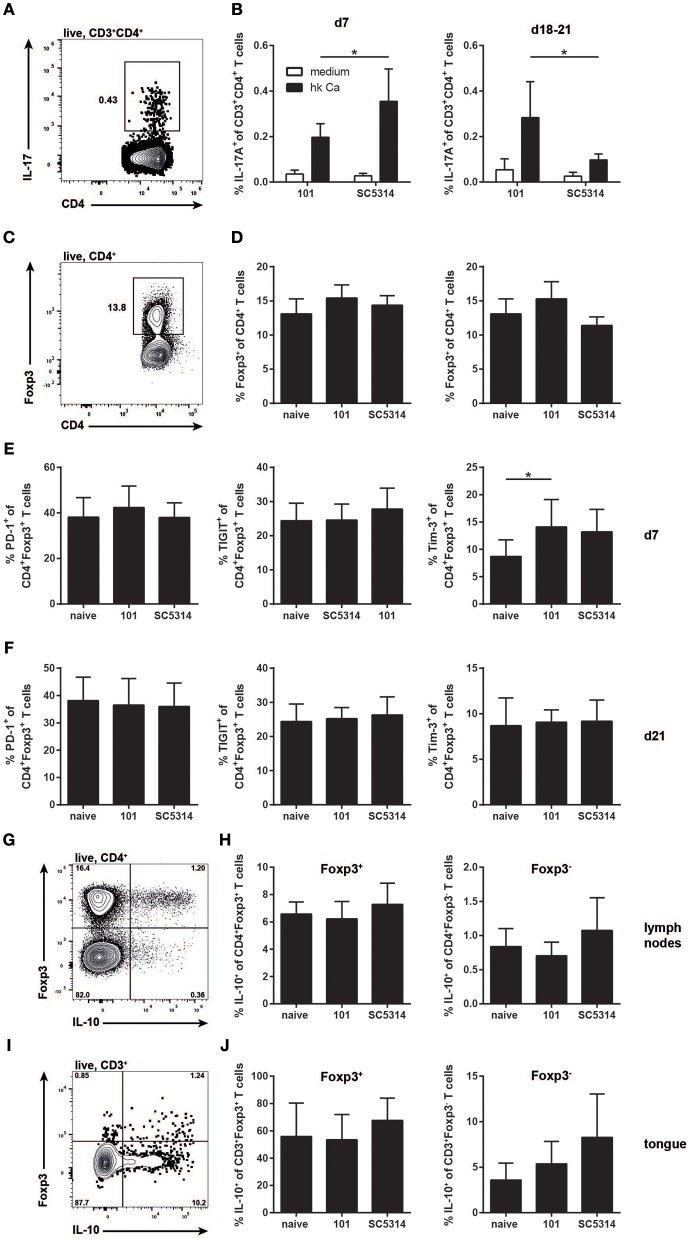
The Treg response during persistent colonization of the oral mucosa with *C. albicans*. **(A–F)** WT mice were sublingually infected with strain 101 or SC5314 and cervical lymph node cells were analyzed on day 7 or day 18–21 as indicated. **(A,B)** Lymph node cells were re-stimulated with MutuDC1940 cells that were pulsed with heat-killed *C. albicans* or left unpulsed for 5 h in the presence of Brefeldin A. IL-17A production by CD3^+^CD4^+^ cells was analyzed by intracellular cytokine staining and flow cytometry. **(C,D)** The frequency of Foxp3-expressing cells within the CD4^+^ lymphocyte compartment was assessed by flow cytometry. **(E,F)** PD-1, TIGIT, and Tim-3 expression by CD4^+^Foxp3^+^ Treg cells was analyzed by flow cytometry. (**G–J**) *Il10*-Thy1.1 reporter mice were sublingually infected with strain 101 or SC5314 or left naive. IL-10 expression by Foxp3^+^ Tregs and Foxp3^−^ effector T cells was assessed in the cervical lymph nodes **(G,H)** and in the tongue **(I,J)** on day 21 post-infection by flow cytometry. Cells were pregated on CD90^+^CD4^+^ **(G,H)** or on CD45.2^+^CD3^+^ **(I,J)**, respectively. Representative FACS plots are shown in (A, C, G, I); summary plots with data pooled from at least 2 experiments with 6–9 animals per infected group and 3–4 animals per naive group are shown in (B, D–F, H, J), with the exception of the right plot in **(D)**, where data are from a single experiment with 3 animals per group. In B, Statistics were calculated using *t*-test. In **(D–F, H, J)**, statistics were calculated using one-way ANOVA. *p < 0.05.

Albeit with delay and more restricted in comparison to strain SC5314, *C. albicans* strain 101 does induce a pronounced immune response in the host, characterized by a prominent IL-17 signature. While induction of the type 17 response does not lead to clearance of strain 101 from the oral mucosa, IL-17 signaling is essential for controlling persistence and preventing fungal overgrowth (12). Importantly, unlike infection with strain SC5314, the type 17 response to strain 101 infection is maintained over a prolonged period of time (Figures 4A,B). Given the potential of type 17 immunity to cause immunopathology and provoke tissue damage (35), tight regulation by mechanisms such as those mediated by Tregs might be necessary.

We thus turned to the analysis of Tregs at later time points of infection to assess their role during persistent colonization of the oral mucosa with *C. albicans*. Treg numbers did not change significantly over the course of 3 weeks of infection and there was also no difference in Treg frequencies between strain 101 and strain SC5314-infected mice (Figures 4C,D). Treg frequencies remained unchanged even after 100 days of *C. albicans* colonization with strain 101 (data not shown). We also examined possible functional differences in Tregs in the different settings. Tregs are characterized by the expression of co-inhibitory receptors. We have shown previously that Tim-3 expression is induced on Tregs in a type 17-polarized environment (36) and this was also observed here (Figure 4E). Differences in Tim-3 expression by Tregs were detected on day 7 post-infection, but were lost at later time points (Figures 4E,F). The expression of other co-inhibitory receptors remained unchanged during the course of OPC with either strain of *C. albicans* (Figures 4E,F).

Next we assessed IL-10 expression by Tregs, a key effector molecule of these cells with a central role in preventing inflammation-mediated tissue damage. IL-10 is very difficult to track by intracellular staining and flow cytometry. Therefore, we made use of *Il10*-Thy1.1 reporter mice (18). These mice also contain a GFP-reporter for Foxp3 (19), making the need for intranuclear staining of Foxp3 dispensable. Infecting these mice with *C. albicans* revealed a strong expression of IL-10 by Foxp3^+^ cells and to a lesser degree by Foxp3^−^ cells (Figures 4G,H). However, again no significant differences in IL-10 production were observed between Tregs from naïve and infected animals, if anything, there was a trend toward higher IL-10 in SC5314 infected mice (Figures 4G,H).

Next, we analyzed IL-10 production by Tregs in the tongue. A prominent fraction of those also expressed IL-10, as assessed by the Thy1.1 reporter (Figures 4I,J). In addition, we detected some IL-10 expression by Foxp3-negative T cells. Albeit with some variation, these populations were detected in all samples analyzed, including the naïve tongue and that of animals infected with either strain of *C. albicans* (Figures 4I,J).

### Tregs and IL-10 Are Not Required for *C. albicans* Persistence in the Murine Oral Mucosa

**Figure 5:**
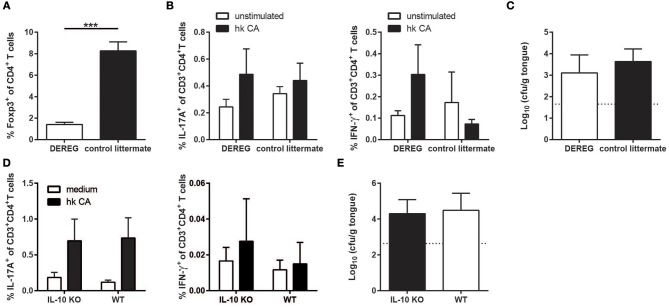
The absence of Tregs or IL-10 does not compromise persistence of strain 101 in the oral epithelium. **(A–C)** DEREG mice and control littermates were sublingually infected with *C. albicans* strain 101 and treated with diphtheria toxin on day 11 and 13 after the antifungal response was fully established. One day after the last treatment, the mice were sacrificed for analysis. **(A)** Treg depletion efficiency was analyzed in the cervical lymph nodes by flow cytometry. Data are the % of Foxp3^+^ cells within the population of CD4^+^ viable cells. **(B)** Lymph node cells were re-stimulated with MutuDC1940 cells that were pulsed with heat-killed *C. albicans* or left unpulsed for 5 h in the presence of Brefeldin A. IL-17A (left) and IFN-γ (right) production by CD3^+^CD4^+^ cells was analyzed by intracellular cytokine staining and flow cytometry. **(C)** The fungal burden was determined by plating tongue homogenates on YPD agar. Each bar represents the mean + SD of 3 to 4 mice per group. Data are from one out of two independent experiments. **(D,E)** IL-10-deficient mice and WT controls were sublingually infected with *C. albicans* strain 101 and analyzed on day 9 post-infection. **(D)** Lymph node cells were re-stimulated and analyzed for IL-17 (left) and IFN-γ (right) production as in B. **(E)** Tongue fungal burdens were analyzed as in C. Each bar represents the mean + SD of 8–9 mice per group pooled from two independent experiments. Statistics were calculated using unpaired t-Test. ***p < 0.001.

Although we did not observe significant changes in the Treg compartment during *C. albicans* colonization of the oral mucosa, the Tregs present might still modulate the antifungal host response to *C. albicans* and thereby contribute to fungal persistence in the oral mucosa. We therefore examined DEREG mice (20) in which Tregs can be ablated by means of diphtheria toxin injection. We chose to initiate Treg depletion at a time point when stable fungal colonization and burden-regulating IL-17 immunity was already established (Figure 5A). To our surprise, we did not observe any significant changes in the degree and the quality of the effector T cell response to *C. albicans* (Figure 5B) or in the growth of the fungus on the mucosa (Figure 5C) upon Treg depletion.

Because IL-10 production was not limited to the Treg compartment, we also assessed the impact of IL-10 itself on the antifungal response and on *C. albicans* colonization levels separately by means of IL-10-deficient mice (21). Similarly to what we observed under Treg-depleting conditions, IL-10 deficiency did not affect the extent or the quality of the *C. albicans*-specific Th17 response in animals infected with strain 101 (Figure 5D). Consistent with this, fungal loads in the tongue were also unchanged in IL-10 knockout mice compared to WT controls (Figure 5E). Together, these findings indicate that immune regulation by Tregs and IL-10 is dispensable for persistent *C. albicans* colonization of the oral mucosa.

## Discussion

Immunoregulatory mechanisms such as those mediated by Tregs and IL-10 are essential for maintaining tolerance and preventing excessive immune responses to commensal microbes. In this study we analyzed the role of immune regulation during *C. albicans* colonization. Our data indicate that fungal persistence in the oral mucosa is not associated with immunosuppression that would blunt a strong host response and prevent rapid fungal elimination at the onset of infection, as it is observed during OPC with the highly virulent strain SC5314. Rather, the failure of strain 101 to induce an early inflammatory response appears to result from intrinsic properties of the strain and how it interacts with the host and in particular with the epithelium.

The epithelial response to *C. albicans* in turn instructs the extent of neutrophil trafficking to the site of infection (39). The fungal determinants that are responsible for the strain-specific differences at the interface with the epithelium and that underlie the differential behavior in contact with the host may comprise differences in filamentation (40), tissue invasion (12), and the production of virulence factors such as the recently identified peptide toxin candidalysin (15). Candidalysin has been linked to mucosal immune activation and neutrophil recruitment (15, 41) with an important contribution of IL-1 family cytokine-dependent chemokine induction (39, 42, 43). Our previous work has shown reduced expression of the ECE1 gene (which encodes for candidalysin) by strain 101 in contact with epithelial cells (12), and this difference to strain SC5314 may at least in part be responsible for the observed interspecies differences. The identification of relevant fungal factors underlying the differential host response is subject of ongoing research.

Induction of an inflammatory response by the highly virulent *C. albicans* strain SC5314 in epithelial cells and tissues has been observed in previous transcriptomic studies (44–47). However, our study is the first, to our knowledge, that assesses the kinetics of the transcriptional response to two functionally distinct *C. albicans* isolates in the murine oral mucosa *in vivo*. While host genes associated with the neutrophil response are differentially regulated during infection with strains SC5314 and 101, many other immune genes including those linked to the IL-17 pathway, are expressed to comparable levels in response to the two *C. albicans* strains under investigation, albeit with delay in case of strain 101. By day 3, IL-17 and related cytokines are expressed at comparable levels in both SC5314- and 101-infected tongues. Similarly, Th17 differentiation was indistinguishable after day 7 post-infection. The differential activation of the epithelial-inflammatory response and the IL-17 pathway by strain 101 underlines the notion that the two host pathways constitute two distinct modules of the antifungal response in the oral mucosa that are regulated largely independently. It also supports our earlier observation that the recruitment of neutrophils to the infected epithelium is not compromised in absence of IL-17 signaling (33), and strong IL-17 induction in response to strain 101 on the other hand is not sufficient for bringing neutrophils to the site of infection (12).

The uncoupling of the IL-17 pathway from the epithelial/neutrophil response is also consistent with our recent finding that induction of IL-17 is instructed by dendritic cells and in particular by the Langerin^+^ subset of dendritic cells that have the unique property in the *C. albicans*-infected oral mucosa to co-produce the three major IL-17-instructing cytokines IL-23, IL-1, and IL-6 (37). The IL-17 response may be further modulated via epithelial sensing of *C. albicans* and IL-1 family cytokines that are released from the epithelium in response to candidalysin in case of infection with strain SC5314 (42, 43).

β-defensin-3 is a prominent target gene of IL-17 and it was proposed to act as the major antifungal effector molecule during OPC (48). Strong induction in response to both strains SC5314 and 101 was confirmed in our RNA-seq analysis. However, surprisingly, β-defensin-3 expression was delayed when compared to the kinetics of IL-17 induction in case of SC5314. It reached its maximum levels only by day 3 post-infection when the fungus was already nearly cleared from the oral mucosa. Together with the observation that the lack of β-defensin-3 resulted in a slightly less severe loss of fungal control than IL-17 signaling deficiency (48), this suggests that additional IL-17-dependent antimicrobial effector molecules are likely involved.

Based on the observation that the IL-17 pathway is crucial for regulating fungal growth independently of the fungal isolate (12), it is surprising that strain 101 is able to resist the continuously activated IL-17-mediated immunity and to persist in the oral mucosa. Its predominant localization to the stratum corneum may limit its exposure to host immune effectors. Further, it is surprising that the prolonged IL-17 response does not trigger signs of tissue damage despite the strong pro-inflammatory potential of the cytokine. The IL-17 pathway can drive severe immunopathology in barrier tissues under certain conditions, as seen for instance in the skin of psoriatic patients and in murine models of psoriasis (49, 50). Th17 cells have been divided in different subtypes that differ in their pathogenic potential (51). Here, we show that a small proportion of all T helper cells in the lymph nodes and in the tongue of mice infected with strain SC5314 or 101 do express IL-10. However, a comprehensive analysis of the cytokine profile of *C. albicans*-specific Th17 cells during persistent and acute OPC remains to be determined in the future.

The generation of Th17 cells was proposed to be supported by Tregs. This might be through the provision of TGF-β (19, 52, 53), but also through the consumption of IL-2, which negatively regulates Th17 differentiation (54). As such, Pandiyan et al. showed in an adoptive transfer system that co-transfer of Tregs with effector T cells enhances Th17 differentiation in response to *C. albicans* (55). In our study, depletion of Tregs in wild type mice did not affect the induction of *C. albicans*-specific Th17 cells, nor did it affect already established type 17 immunity when Tregs were depleted at a later time point of persistent colonization. Similar results were also obtained in mice lacking IL-10, one of the key regulatory cytokines and a potent suppressor of Th17 cells (56). Together, our data thus indicate that immune regulation does not make an essential contribution to the balance of a protective non-pathological Th17 response and fungal persistence in our model.

In conclusion, our study indicates that the attenuated host response to *C. albicans* strain 101 is well-tared to allow fungal persistence in the oral mucosa by preventing elimination of the fungus but also uncontrolled fungal overgrowth as well as avoiding the development of immunopathology.

## Ethics Statement

All mouse experiments described in this study were conducted in strict accordance with the guidelines of the Swiss Animal Protection Law and were performed under protocols approved by the Veterinary office of the Canton Zurich, Switzerland (license number 201/2012 and 183/2015). All efforts were made to minimize suffering and ensure the highest ethical and human standards.

## Author Contributions

FRK, KL, SA, NJ, and SL-L: conceptualization; SA and DS: methodology; FRK, KL, SA, FS, and CL: investigation; FRK, KL, SA, VDTT, FS, and CL: formal analysis; NJ, DS, and SL-L: validation and project administration; FRK, KL, VDTT, and SL-L: visualization; MP, NJ, DS, and SL-L: funding acquisition; MP, NJ, and SL-L; supervision; FRK and SL-L: writing-original draft; KL, SA, VDTT, NJ, and DS: writing-review and editing.

### Conflict of Interest Statement

SA currently works for Novartis but on a different topic and the data are restricted to his previous role in the LeibundGut-lab. The remaining authors declare that the research was conducted in the absence of any commercial or financial relationships that could be construed as a potential conflict of interest.

## Supplementary Material

Figure S1 Characterization of tongue epithelial sheets. Epithelial sheets were isolated from infected tongues as described in the Methods and analyzed by microscopy after periodic acid-Schiff staining **(A)** or digested with trypsin to obtain single cell suspensions and analyzed by flow cytometry **(B)** for their composition. In **(A)**, the black arrow indicates epithelial cells; the white arrow indicates cellular infiltrates; the white arrow head indicates *C. albicans* hyphae. The numbers indicate the % of cells in each gate. The representative data shown are from strain SC5314-infected animals.

Figure S2 Overlap of differentially expressed genes at different time points and by the different strains of *C. albicans*. Venn diagrams showing the number of differentially expressed genes in tongue epithelial sheets at each time point after infection with *C. albicans* strain SC5314 **(A)** or strain 101 **(B)**. **(C)** Venn diagram showing the differentially expressed genes on day 1 and 3 after infection with strain SC5314 and on day 3 and 7 after infection with strain 101, respectively, i.e., the conditions with the highest number of gene expression activity. The numbers in the overlapping areas refer to the number of genes that were co-regulated in the respective conditions.

Figure S3 The host response to *C. albicans* in the tongue is dominated by changes in the immune response and metabolic processes. The heat maps show the upregulated **(A)** and downregulated **(B)** GO processes with the smallest FDR. The heat map was arranged by hierarchical clustering using the distance of one minus Pearson correlation and the average linkage mode.

Figure S4 Transcriptional response to *C. albicans* strain 101 during persistent oral infection. **(A,B)** Mice were infected with strain 101 for 60 days. Relative expression of *Cxcl1* (left), *Cxcl2* (middle), and *Cxcl5* transcripts (right) in the bulk tongue tissue **(A)** and cfu in the feces **(B)** are shown. Each symbol represents one animal. The geomean of each group is indicated. **(C–E)** Mice were infected with strain 101 for 12–14 months. The fungal load in the feces **(C)** and in the tongue **(D)** are shown. **(E)** shows representative tongue histology stained with PAS from one of the still highly colonized mice. **(F)** Relative expression of *S100a8* (left), *Lcn2* (middle), and *Defb1* transcripts (right) in the tongue of mice were infected with strains 101 or SC5314 for the indicated period of time. Each symbol represents one animal. The geomean of each group is indicated. Dotted line: geomean of a naive control group. Data are pooled from two independent experiments each, except for fecal cfu in **(C)**, which are from one experiment only. Statistics were calculated using one-way ANOVA (comparison of infected to naïve groups). ^*^*p* < 0.05, ^**^*p* < 0.01, ^***^*p* < 0.001, ^****^*p* < 0.0001.

Figure S5 Treg Depletion. % Foxp3^+^ cells within the splenic CD4^+^CD3^+^ population of mice treated with anti-CD25 antibody or isotype control as indicated.

Table S1 Oligonucleotides used in this study.

Table S2 Antibodies used in this study.
