# Supplementary figures and images for: Persistence of Candida albicans in the Oral Mucosa Induces a Curbed Inflammatory Host Response That Is Independent of Immunosuppression
Source: Front Immunol. 2019 Feb 27;10:330. doi: 10.3389/fimmu.2019.00330 (PMC6400982; doi:10.3389/fimmu.2019.00330)

Fig. S1

A

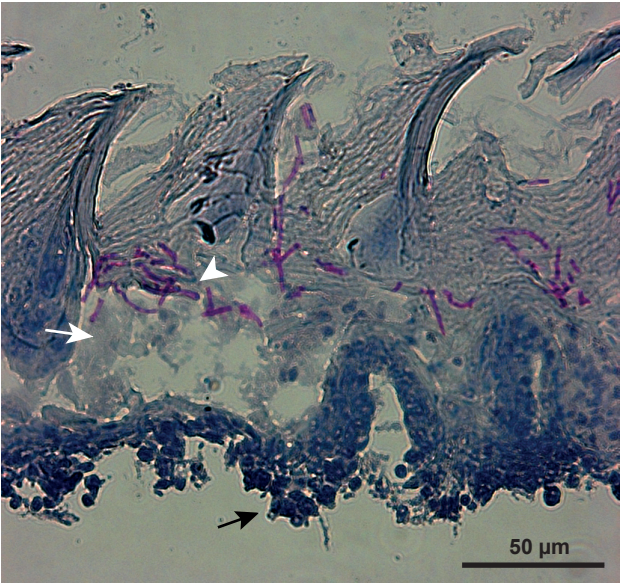

B

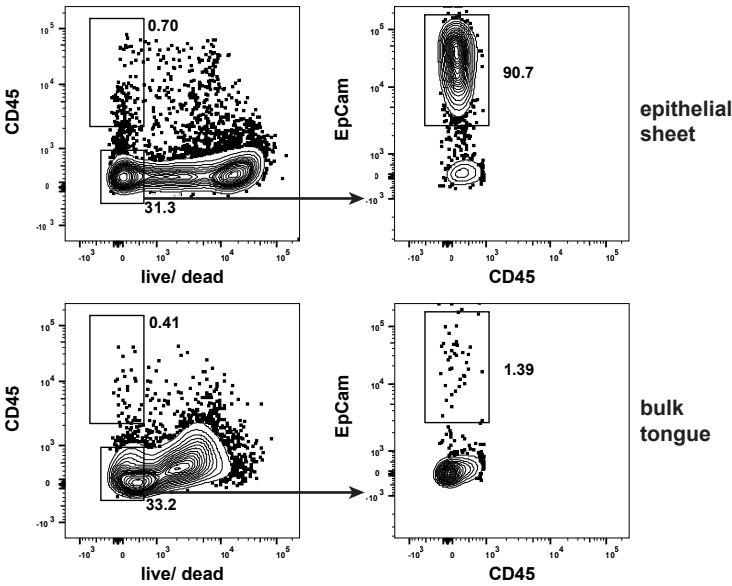

**Fig. S2**

**A**

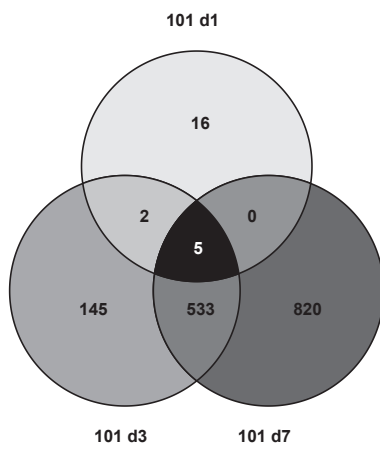

**B**

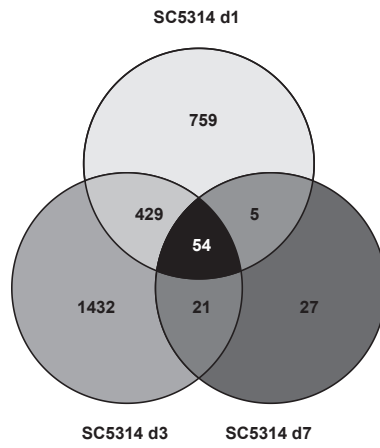

**C**

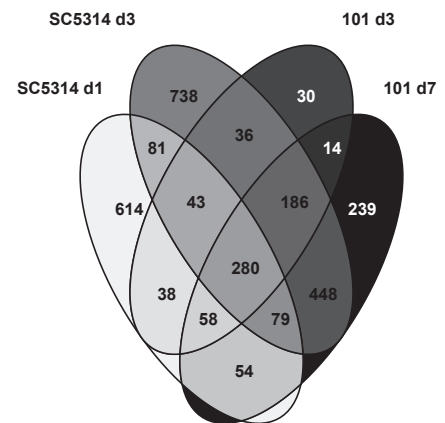

# A

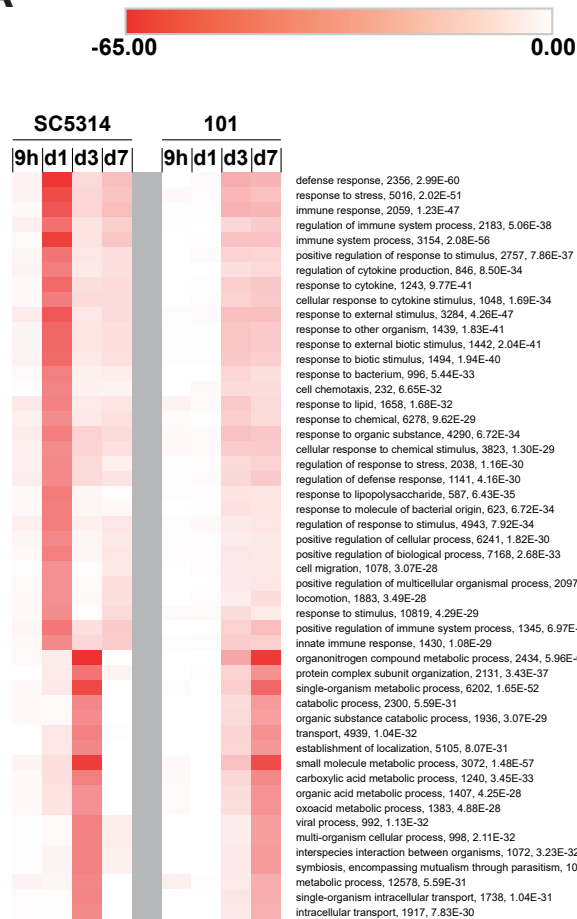

# B

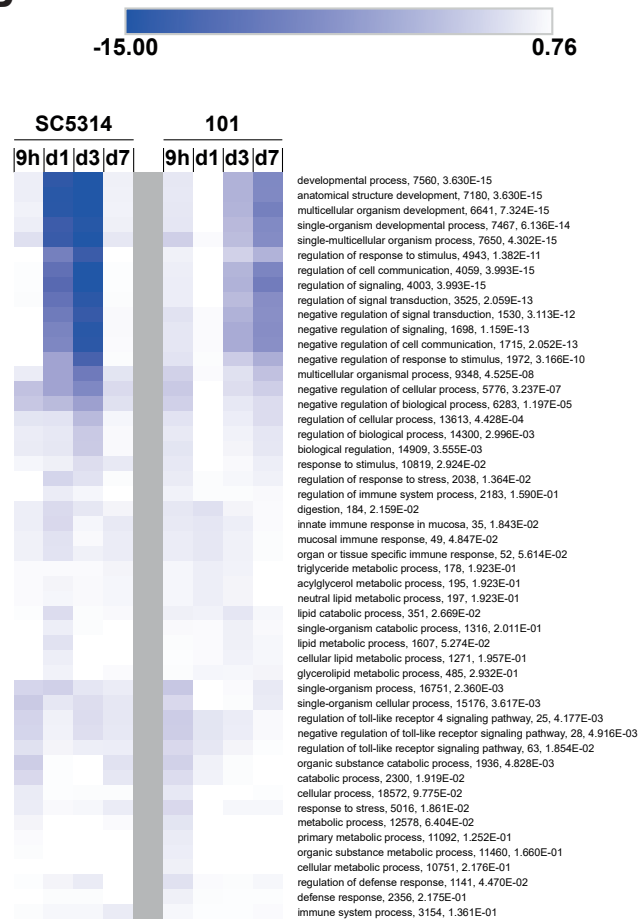

Fig S4

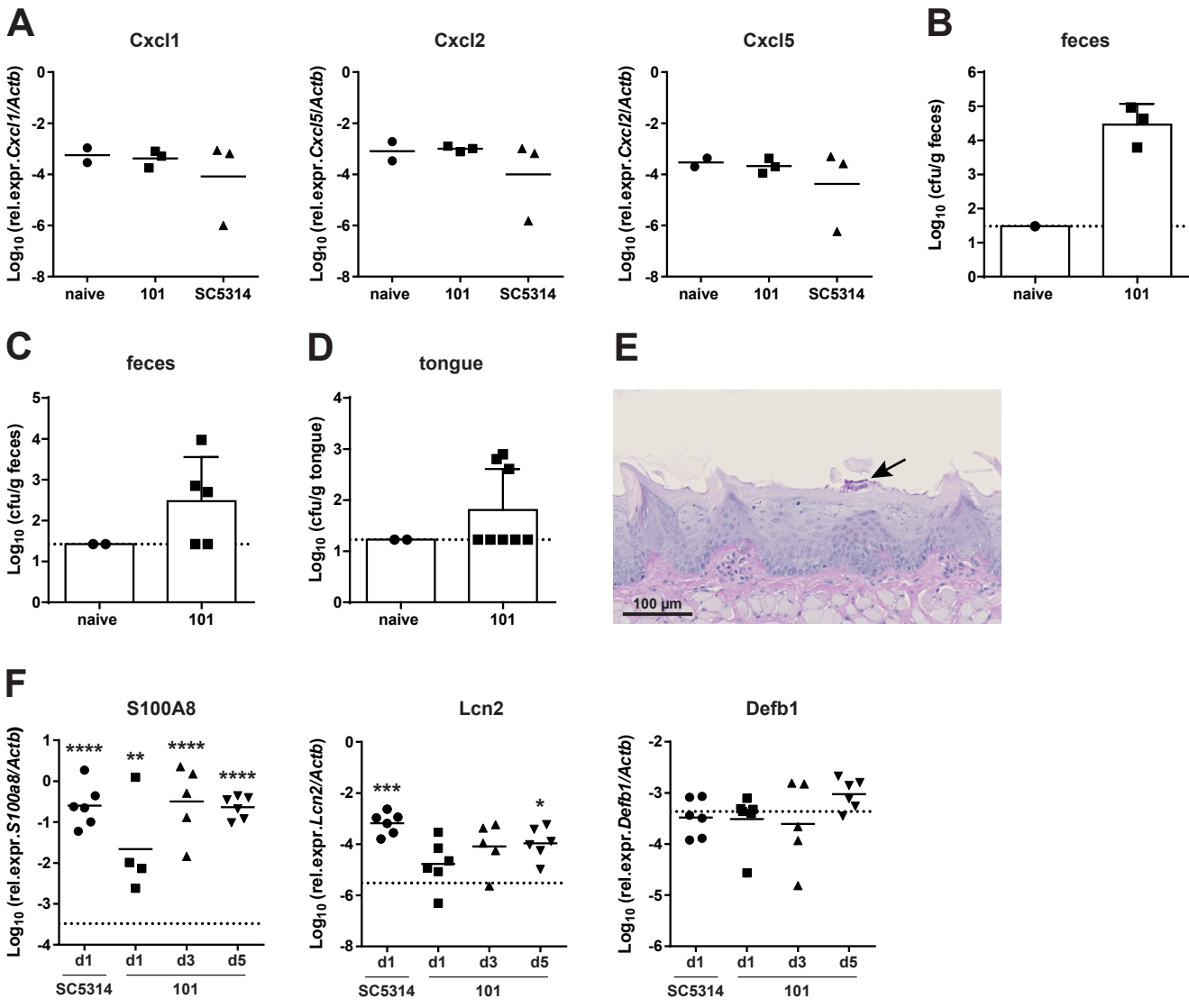

Fig. S5

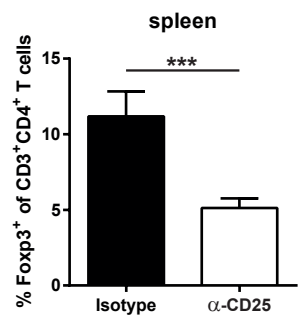

Supplement: Figure S1 — Characterization of tongue epithelial sheets. Epithelial sheets were isolated from infected tongues as described in the Methods and analyzed by microscopy after periodic acid-Schiff staining (A) or digested with trypsin to obtain single cell suspensions and analyzed by flow cytometry (B) for their composition. In (A), the black arrow indicates epithelial cells; the white arrow indicates cellular infiltrates; the white arrow head indicates C. albicans hyphae. The numbers indicate the % of cells in each gate. The representative data shown are from strain SC5314-infected animals. [file Data_Sheet_1.PDF]
