# Supplementary material for: Persistence of Candida albicans in the Oral Mucosa Induces a Curbed Inflammatory Host Response That Is Independent of Immunosuppression
Source: Front Immunol. 2019 Feb 27;10:330. doi: 10.3389/fimmu.2019.00330 (PMC6400982; doi:10.3389/fimmu.2019.00330)
Supplement: Table S1 — Oligonucleotides used in this study. [file Table_1.docx]

Table S1: Oligonucleotides used in this study

| **Oligonucleotides** | **Source** | **Identifier** |
| --- | --- | --- |
| *Actb:* fwd 5´-CCC TGA AGT ACC CCA TTG AAC-3´, rev 5´-CTT TTC ACG GTT GGC CTT AG-3´ | Microsynth | [12] |
| *Tgfb1*: fwd 5´-CAA CAA TTC CTG GCG TTA CCT TGG-3´, rev 5´- GAA AGC CCT GTA TTC CGT CTC CTT-3´ | Microsynth | [57] |
| *Il10*: fwd 5´-GGT TGC CAA GCC TTA TCG GA-3´, rev 5´-ACC TGC TCC ACT GCC TTG CT-3´ | Microsynth | [58] |
| *Cxcl1*: fwd 5´-CCG CTC GCT TCT CTG TG-3’, rev 5’-GCA GCT CAT TGG CGA TAG-3’ | Microsynth | [33] |
| *Cxcl2*: fwd 5´-AGT GAA CTG CGC TGT CAA TGC-3’, rev 5’-GCA AAC TTT TTG ACC GCC CT-3’ | Microsynth | [12] |
| *Cxcl5*: fwd 5´-GAA AGC TAA GCG GAA TGC AC-3’, rev 5’-GGG ACA ATG GTT TCC CTT TT-3’ | Microsynth | N/A |
| *Il17a*: fwd 5´-gct cca gaa ggc cct cag a-3’, rev 5’-agc ttt ccc tcc gca ttg a-3’ | Microsynth | [58] |
| *Il17f*: fwd 5´-GAG GAT AAC ACT GTG AGA GTT GAC -3’, rev 5’-GAG TTC ATG GTG CTG TCC -3’ | Microsynth | [59] |
| *S100a8*: fwd 5´-ATC ACC ATC GCA AGG AAC TC-3’, rev 5’-CCA TGC CCT CTA CAA GAA TG-3’ | Microsynth | N/A |
| *S100a9*: fwd 5´-GTC CAG GTC CTC CAT GAT GT-3’, rev 5’-TCA GAC AAA TGG TGG AAG CA-3’ | Microsynth | N/A |
| *Lcn2*: fwd 5´-AAT GTC ACC TCC ATC CTG GT-3’, rev 5’-CCC TGG AGC TTG GAA CAA AT-3’ | Microsynth | [60] |
| *Defb1*: fwd 5´-CTG GGA GTT TCA CAT CCT CTC-3’, rev 5’-CTC CAT GTT GAA GGC ATT TGT-3’ | Microsynth | [61] |

Table S2: Antibodies used in this study

| **Antibodies (clone)** | **Source** | **Identifier** |
| --- | --- | --- |
| Ax488 anti-GFP | Invitrogen | Cat#A-21311 |
| FITC anti-mFoxp3 (FJK-16s) | eBioscience™ | Cat#11-5773-82 |
| PE anti-mCD45.2 (104) | Biolegend | Cat#109808 |
| PE anti-Ep-CAM (G8.8) | Biolegend | Cat# 118206 |
| PE/Dazzle 594 anti-mTCRβ (H57-597) | Biolegend | Cat#109240 |
| PE/Cy5 anti-mCD3e (145-2C11) | Biolegend | Cat#100310 |
| PE/Cy5 anti-mCD45.2 (30-F11) | Biolegend | Cat#103109 |
| Pe/Cy7 anti-mIL-17A (TC11-18H10.1) | Biolegend | Cat#506921 |
| APC anti-mIFN-γ (XMG1.2) | Biolegend | Cat#505810 |
| PB anti-mCD4 (RM4-5) | Biolegend | Cat#100531 |
| BV421 anti-mIFN-γ (XMG1.2) | Biolegend | Cat#505830 |
| BV570 anti-mCD90.2 (30-H12) | Biolegend | Cat#105329 |
| BV605 anti-mCD90.1 (OX7) | Biolegend | Cat#202537 |
| BV605 anti-mCD4 (RM4-5) | Biolegend | Cat#100548 |
| APC anti-mFoxp3 (FJK-16s) | eBioscience™ | Cat#17-5773-82 |
| Biotin anti-mPD1 (J43) | eBioscience™ | Cat#13-9985-85 |
| PE anti-mTIGIT (1G9) | BioLegend | Cat#142104 |
| PE anti-mTim 3 (#215008) | R&D Systems | FAB1529P |
| SB600 anti-mCD4 (RM4-5) | eBioscience™ | Cat#63-0042-82 |
| Biotin anti-mCD3 (17A2) | BioLegend | Cat#100244 |
| APC anti-mCD45.2 (104) | BioLegend | Cat#109814 |
| BV650 anti-mCD90.1 (OX-7) | BioLegend | Cat#202533 |
| BV421 anti-mCD90.1 (OX-7) | BioLegend | Cat#202529 |
| PerCP anti-mCD90.2 (30-H12) | BioLegend | Cat#105322 |
| Zombie NIR Fixable Viability Kit | BioLegend | Cat#423106 |
